# Anterior Shoulder Dislocation With an Ipsilateral Humeral Shaft Fracture: A Case Report

**DOI:** 10.7759/cureus.33307

**Published:** 2023-01-03

**Authors:** Abdulmalik B Albaker, Ahmad Abdullah A Alsaleh, Mishari Malik Alshammari, Hatim Abdullah Akkasi, Hazzaa Abdullah Hazza Alharbi, Norah Ibrahim S Alqurmulah

**Affiliations:** 1 Orthopaedic Department, College of Medicine, Majmaah University, Majmaah, SAU; 2 Orthopaedic Department, King Saud Hospital, Unaizah, SAU; 3 Family Medicine Department, Ain Dar Primary Healthcare Center (PHC), Ain Dar, SAU; 4 Emergency Department, Khamis Mashit Hospital, Khamis Mashit, SAU; 5 Clinical Sciences Department, Majmaah University, Majmaah, SAU; 6 Ophthalmology Department, King Saud Hospital, Unaizah, SAU

**Keywords:** intramedullary nail, radial nerve injury, road traffic injuries, shoulder anterior dislocation, midshaft humerus fractures

## Abstract

Anterior shoulder dislocation is the most common type of shoulder dislocation but if accompanied by an ipsilateral humeral shaft fracture, it becomes extremely rare. There was no clear approach for dealing with these cases. We would like to present a case of a 17-year-old medically free male who was brought to the emergency department by ambulance after a road traffic accident. The patient was conscious, alert, and oriented. His Glasgow Coma Scale (GCS) was 15/15. He had multiple bruises all over his body with obvious swelling in his right arm with an inability to move the arm. There was tenderness over the right arm but an intact distal neurovascular exanimation. X-ray and CT scan showed anterior shoulder dislocation with an ipsilateral humeral shaft fracture of the right arm. There is no specific approach for such cases. However, open reduction with an intramedullary nail showed good outcomes with fewer postoperative neurovascular complications.

## Introduction

Anterior shoulder dislocation is considered the most common dislocation of the shoulder that presents to the emergency department. The shoulder is composed of the humerus, scapula, and clavicle, and it has four different joints, which are the glenohumeral, acromioclavicular, sternoclavicular, and scapulothoracic joints [1]. The shoulder is stabilized by two types of factors, passive and active. The passive stabilizers are bony geometry, labrum, capsule, and glenohumeral ligaments. The active factors are the biceps, rotator cuff, and deltoid muscle [2]. The humeral shaft is the part between the surgical neck and epicondyle. Humeral shaft fractures account for 3% of all bone fractures [3]. However, a case of anterior shoulder dislocation with an ipsilateral mid-humeral shaft fracture is rare and not commonly seen. High-velocity trauma frequently causes similar cases. Because of this, very few cases were reported globally, which resulted in an unclear approach to such cases [4].

## Case presentation

A 17-year-old male was brought to the emergency department by ambulance after a road traffic accident (RTA). He was sitting in the front seat, wearing a seatbelt. The car did not roll over, and he was not ejected out of the vehicle. He did not remember his exact position inside the car at the time of the accident. In the primary assessment, the patient was conscious, oriented, alert, and hemodynamically stable, and his Glasgow Coma Scale (GCS) was 15/15; however, he was in severe pain. There were multiple minor injuries and bruises on the face, chest, and abdomen, and obvious deformity in the right arm. The patient complained of right arm pain with tenderness and inability to move the right arm. On examination, the patient looked unwell and distressed. There was swelling in the right arm and pain with minimal passive movement. The neurovascular examination was normal.

**Figure 1:**
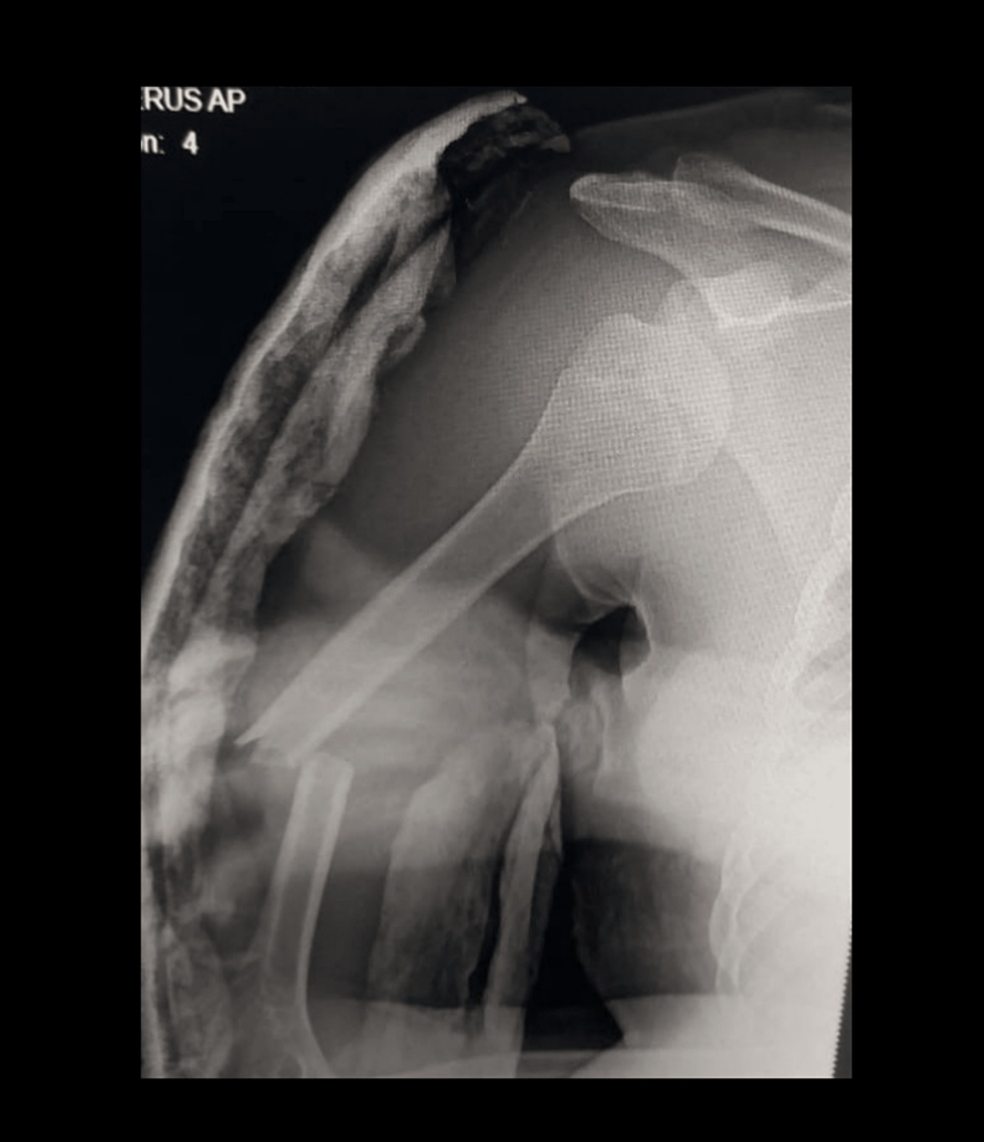
X-ray showing anterior dislocation of the right shoulder with an ipsilateral mid-shaft fracture of the humerus

**Figure 2:**
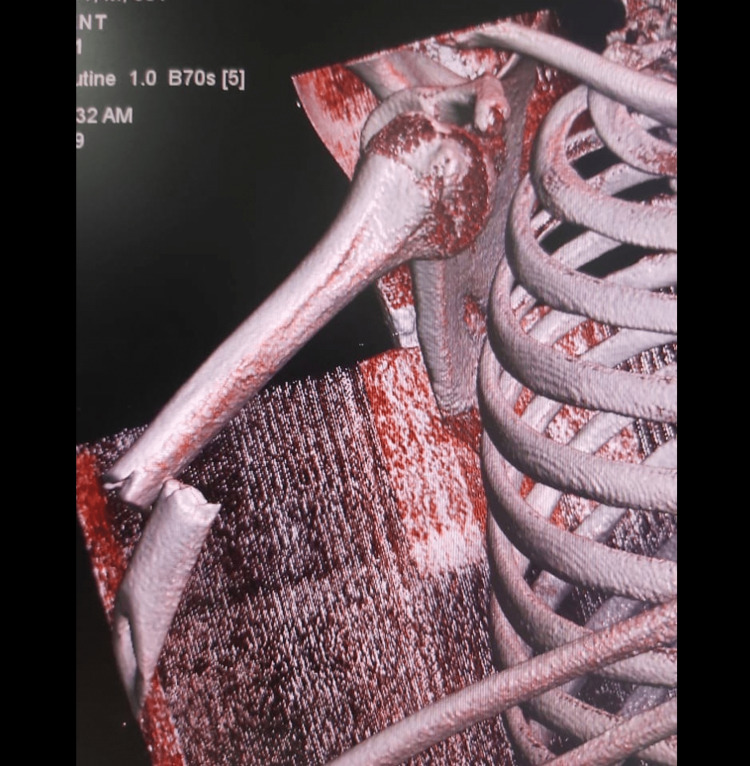
CT scan 3D showing anterior dislocation of the right shoulder with a mid-shaft fracture of the humerus

The initial plain X-ray and CT showed anterior dislocation of the right shoulder with an ipsilateral mid-shaft fracture of the humerus (Figures 1, 2).

**Figure 3:**
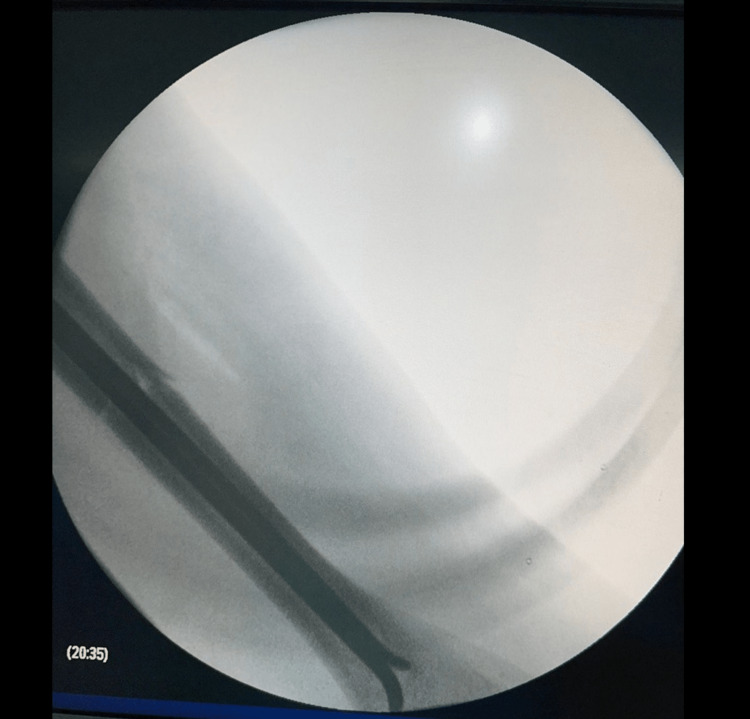
Intraoperative X-ray with intramedullary nails

**Figure 4:**
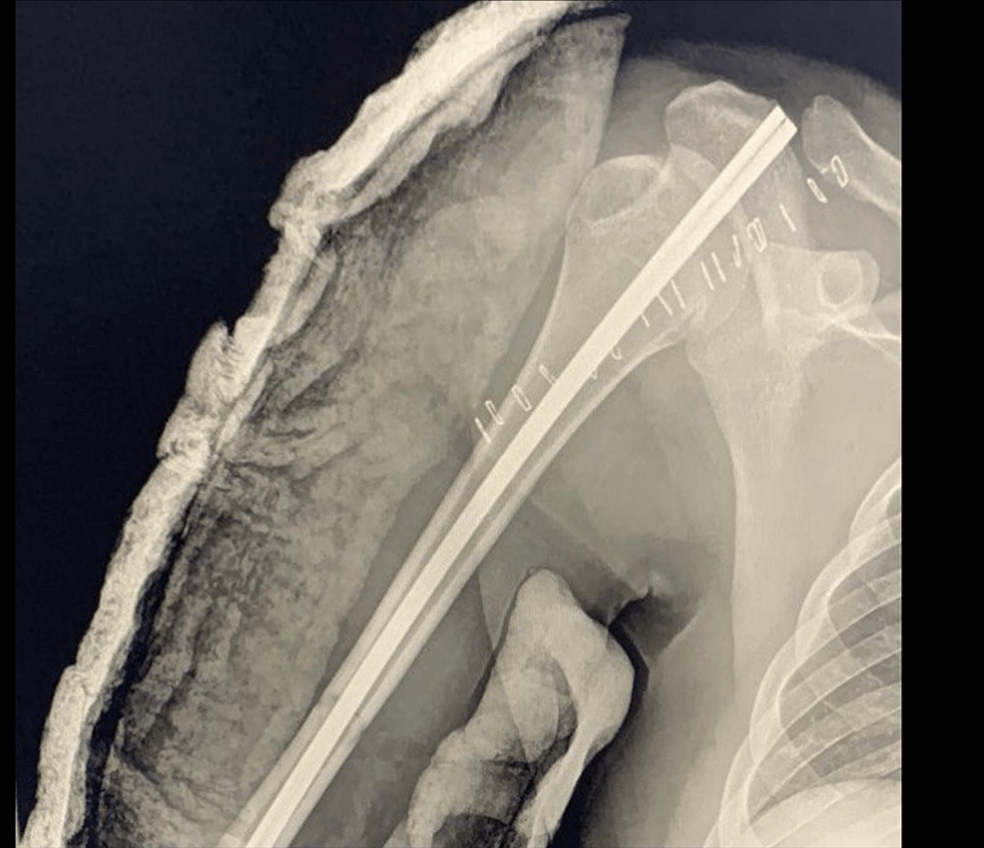
Postoperative X-ray

Urgent closed reduction under surgical sedation was done by direct manipulation of the humeral head but the reduction failed. Due to the failure of closed reduction, the patient was shifted to the operating room (OR) the same night, as an open reduction was indicated. Intraoperatively, we reached the shoulder through a deltopectoral approach. Initially, shoulder dislocation was reduced easily. We then tried to insert the anterograde guide wire, but it could not pass the fracture site. Then, we tried to do open reduction and exploration of the fracture site but there was a blockage of the medullary cavity by blood clots, which explains the failure of the insertion of the guide wire. The blood clots were evacuated and the medullary cavity was washed. The guide wire was then inserted into the medullary cavity successfully. Immobilization of the limb was done by a U-shaped cast (Figures 3, 4).

**Figure 5:**
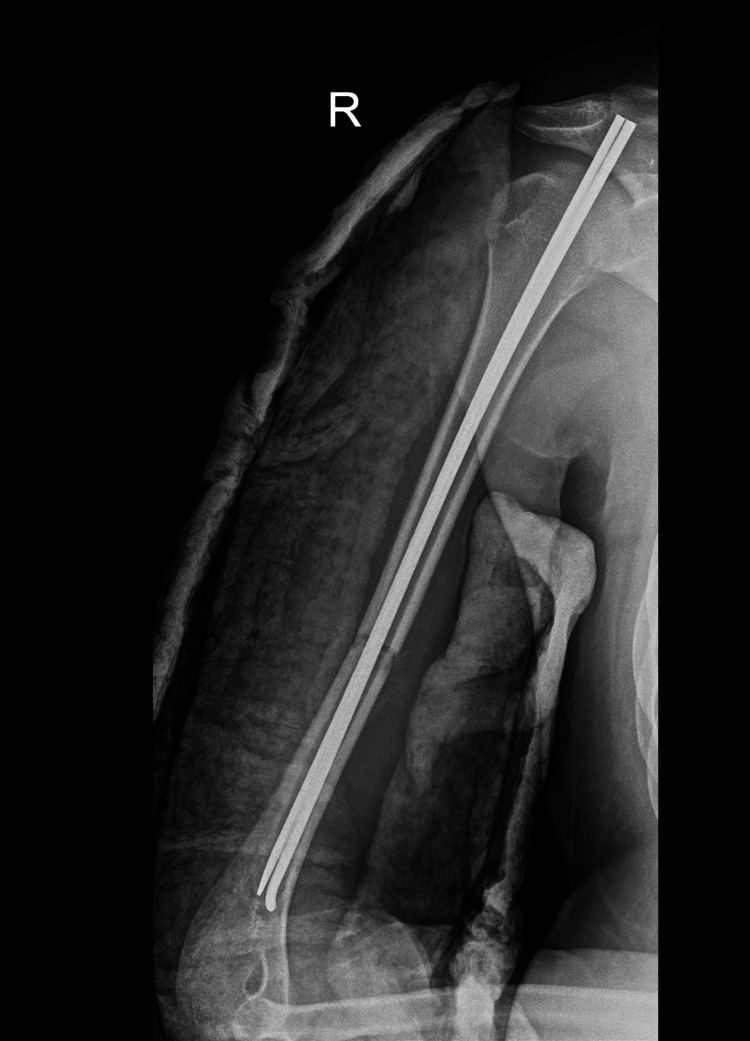
X-ray four weeks post-surgery

There were no problems postoperatively; the patient was looking well, not in distress, and passing urine and stool. There was no nerve injury, especially at the level of the radial nerve. The patient was discharged after three days with follow-up in the outpatient department (OPD). Figure 5 shows the fracture four weeks following discharge.

**Figure 6:**
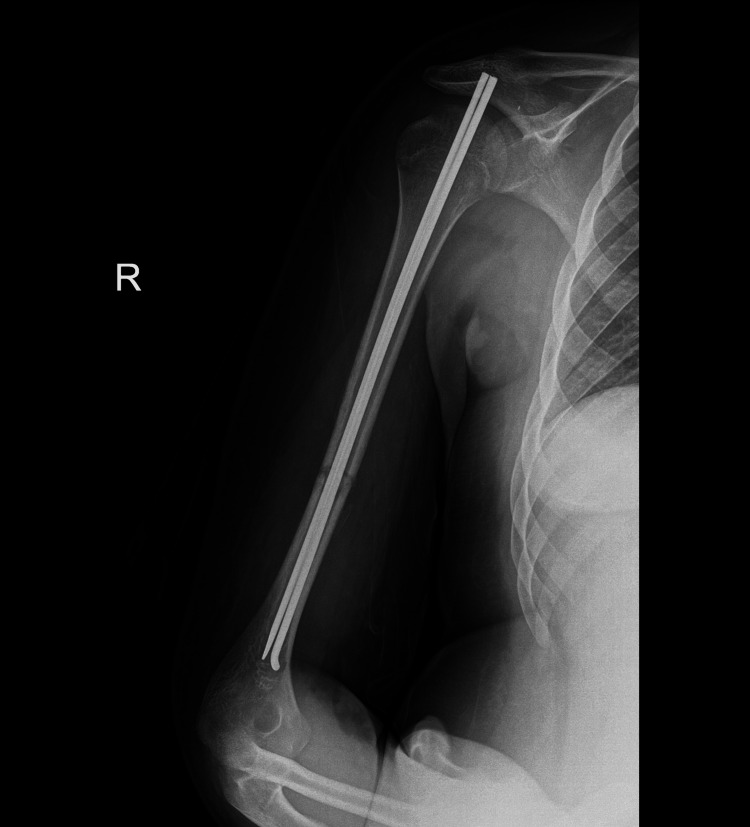
X-ray six weeks post-surgery, with healing signs

After six weeks, the cast was removed with minimal progressive mobilization of the affected limb (Figure 6).

## Discussion

Anterior shoulder dislocation is a common case, but if it is accompanied by an ipsilateral mid-shaft fracture of the humerus, this makes it a rare case. There has been a total of around 30 cases; the first case was reported by Winderman in 1940, and the latest was reported by Hara M and Yamazaki K [5,6].

A similar mechanism to our case was described in dashboard automobile injuries in femoral fractures with an ipsilateral hip dislocation [7]. Sankaran-Kutty described the possible mechanism of the transmission of force through the humeral axis to the shoulder, which results in similar cases [8]. Kontakis GM believes that the dislocation occurs before the fracture [9].

Various treatment techniques were reported; a closed reduction with splinting was successful four of five times [5,9,10]. Closed reduction with external fixation was addressed in two reports [8,11], with good outcomes. Kazakos K believes that in a case of an irreducible anterior shoulder dislocation combined with a humeral fracture, the intramedullary nail has more advantages and better outcomes [12]. Rare cases usually have no direct or clear approach. Since our case is rare, there is a possible general approach or method of treating such cases: (1) Closed reduction of both the dislocation and fracture; (2) Closed reduction of the dislocation with open reduction of the fracture; (3) Closed reduction of the fracture with open reduction of the dislocation; (4) Open reduction of both the dislocation and fracture [13].

Approaching the shoulder first and then the humeral shaft fracture reduction is a better method than reducing the fracture first. In case of successful reduction of the shoulder, we can deal with the humerus either with a surgical or a non-surgical approach [14].

## Conclusions

In summary, shoulder dislocation with a humerus shaft fracture has no straightforward protocol. It differs from one case to another. Closed reduction of both injuries is preferred if it is possible. If closed reduction is not achievable, open reduction with an intramedullary nail is a good option and has a great outcome as we did in this case.
